# The epidemiology of diphtheria in Haiti, December 2014–June 2021: A spatial modeling analysis

**DOI:** 10.1371/journal.pone.0273398

**Published:** 2022-08-22

**Authors:** Juniorcaius Ikejezie, Tessa Langley, Sarah Lewis, Donal Bisanzio, Revati Phalkey

**Affiliations:** 1 Division of Epidemiology and Public Health, School of Medicine, University of Nottingham, Nottingham, United Kingdom; 2 RTI International, Washington, District of Columbia, United States of America; 3 Climate Change and Health Unit, UK Health Security Agency, London, United Kingdom; 4 Heidelberg Institute of Global Health, University of Heidelberg, Heidelberg, Germany; University of Illinois Urbana-Champaign College of Veterinary Medicine, UNITED STATES

## Abstract

**Background:**

Haiti has been experiencing a resurgence of diphtheria since December 2014. Little is known about the factors contributing to the spread and persistence of the disease in the country. Geographic information systems (GIS) and spatial analysis were used to characterize the epidemiology of diphtheria in Haiti between December 2014 and June 2021.

**Methods:**

Data for the study were collected from official and open-source databases. Choropleth maps were developed to understand spatial trends of diphtheria incidence in Haiti at the commune level, the third administrative division of the country. Spatial autocorrelation was assessed using the global Moran’s I. Local indicators of spatial association (LISA) were employed to detect areas with spatial dependence. Ordinary least squares (OLS) and geographically weighted regression (GWR) models were built to identify factors associated with diphtheria incidence. The performance and fit of the models were compared using the adjusted r-squared (R^2^) and the corrected Akaike information criterion (AIC_c_).

**Results:**

From December 2014 to June 2021, the average annual incidence of confirmed diphtheria was 0.39 cases per 100,000 (range of annual incidence = 0.04–0.74 per 100,000). During the study period, diphtheria incidence presented weak but significant spatial autocorrelation (I = 0.18, p<0.001). Although diphtheria cases occurred throughout Haiti, nine communes were classified as disease hotspots. In the regression analyses, diphtheria incidence was positively associated with health facility density (number of facilities per 100,000 population) and degree of urbanization (proportion of urban population). Incidence was negatively associated with female literacy. The GWR model considerably improved model performance and fit compared to the OLS model, as indicated by the higher adjusted R^2^ value (0.28 v 0.15) and lower AIC_c_ score (261.97 v 267.13).

**Conclusion:**

This study demonstrates that GIS and spatial analysis can support the investigation of epidemiological patterns. Furthermore, it shows that diphtheria incidence exhibited spatial variability in Haiti. The disease hotspots and potential risk factors identified in this analysis could provide a basis for future public health interventions aimed at preventing and controlling diphtheria transmission.

## Introduction

Diphtheria is a highly contagious, vaccine-preventable disease caused by *Corynebacterium diphtheriae* [1–3]. Transmission occurs primarily by droplet or contact with nasopharyngeal secretions of infected people. The hallmark of infection is the formation in the upper respiratory tract of the pseudomembrane–a thick, gray coating consisting of necrotic tissue and bacteria [1–3]. Diphtheria complications include respiratory insufficiency, myocarditis, and neuritis. The fatality rate among confirmed cases is 5–10%. However, higher rates have been observed among certain groups (e.g., untreated, unvaccinated individuals) [1–3].

In recent years, despite the existence of a safe and effective vaccine, diphtheria has been experiencing a dramatic resurgence worldwide, with major outbreaks reported in Bangladesh, Venezuela, and Yemen [4–6]. In 2019 alone, 22,625 cases were reported globally–a 407% increase from 2015, when 4,535 infections were recorded [6]. The situation is exacerbated by the current shortage of the life-saving diphtheria antitoxin, resulting from a decline in production due to decreasing demand [7,8].

Presently, Haiti is among the countries worst hit by the disease in the Americas. From December 2014 to June 2021, 1,281 suspected cases were detected in the country [9]. Past research has shown inadequate levels of diphtheria immunization among confirmed cases in Haiti [10–12]. Nevertheless, little is known about other factors contributing to the spread and persistence of the disease in the country. Moreover, areas at high risk for infection remain unknown. Understanding the spatial patterns of diphtheria transmission and the associated factors is critical for developing and implementing effective interventions.

Over the last two decades, geographic information systems (GIS) and spatial analysis have emerged as key tools for detecting disease hotspots and identifying factors correlated with disease transmission [13,14]. Few studies have employed GIS and spatial analysis to examine diphtheria. For instance, Podavalenko [15] detected a significant correlation between diphtheria incidence and vaccination coverage, population density, and population growth rate in Ukraine during 1985–2016. Nailul *et al*. [16] also identified a negative association between diphtheria incidence and vaccination coverage in East Java, Indonesia in 2010. Furthermore, Quesada [17] found that diphtheria incidence was associated with poverty rates during an outbreak in San Antonio, Texas in 1970.

The present study set out to characterize the spatial epidemiology of diphtheria in Haiti from December 2014 to June 2021. Specifically, it aimed to determine the subnational distribution of confirmed cases in the country; locate hotspots of transmission; and identify potential factors associated with the incidence of the disease.

## Methods

### Study area

Haiti (19.00° N latitude, 72.25° W longitude) is situated on the western third of Hispaniola, an island in the Caribbean Sea that it shares with the Dominican Republic [18,19]. It is divided into 10 departments consisting of 42 arrondissements, 140 communes, and 570 communal sections. The capital and largest city is Port-au-Prince, which is in the Ouest department. Haiti’s population is estimated at about 11 million.

### Study design

The study was a retrospective ecological analysis of confirmed diphtheria cases reported to Haiti’s Directorate of Epidemiology, Laboratory and Research (Direction d’ épidémiologie, des laboratoires et de la recherche; DELR)–a body of Haiti’s Ministry of Public Health and Population (Ministère de la santé publique et de la population; MSPP) that records, reviews, and validates data of all diphtheria cases reported in the country. In this study, a confirmed case was defined as an individual who tested positive for *C*. *diphtheriae* by polymerase chain reaction (PCR) or who was confirmed by epidemiological link. The geographical unit of analysis was the commune. The period under consideration was from 1^st^ December 2014 to 30^th^ June 2021.

### Data dictionary

The number of diphtheria cases at the commune level were obtained from the DELR. Crude annual rates by communes were calculated by dividing the number of diphtheria cases reported annually by the corresponding population estimate from the Haitian Institute of Statistics and Informatics (Institut haïtien de statistique et d’informatique; IHSI) [18]. Average rates were calculated by dividing the sum of the total cases reported during the study period by the sum of the populations for the same period. All rates were multiplied by 100,000. Eleven factors which could be linked to diphtheria incidence were selected following a systematic literature review [20]. These were grouped under three domains: health, socioeconomic status, and environment. **Table 1** summarizes the study variables.

**Table 1:** Variables selected for the analysis.

Theme and variable	Description	Source and study period
Diphtheria incidence	Confirmed diphtheria cases per 100,000 population	Directorate of Epidemiology, Laboratory and Research, 2014–21
**Health**		
Coverage for the third dose of the diphtheria tetanus pertussis (DTP3) vaccine	Proportion of children aged <1 year who had received the third dose of the DTP vaccine	Ministry of Public Health and Population, 2015–20
Diphtheria tetanus (DT) vaccine stockout	Average annual number of days when the DT vaccine was out of stock	Ministry of Public Health and Population, 2017–20
DTP stockout	Average annual number of days when the DTP vaccine was out of stock	Ministry of Public Health and Population, 2017–20
Health facility density	Number of health facilities per 100,000 population	Humanitarian Data Exchange, 2020 [21]
**Socioeconomic status**		
Female literacy	Proportion of women who are literate	Demographic and Health Surveys Program, 2016–17 [22]
Improved water source	Proportion of the population that lives in households whose main source of drinking water is an improved source	Demographic and Health Surveys Program, 2016–17 [22]
Male literacy	Proportion of men who are literate	Demographic and Health Surveys Program, 2016–17 [22]
No toilet facility	Proportion of the population that lives in households with no toilet facility	Demographic and Health Surveys Program, 2016–17 [22]
School density	Education facilities per 100,000 population	Demographic and Health Surveys Program, 2020 [22]
**Environment**		
Population density	Ratio between total population and total surface area	Haitian Institute of Statistics and Informatics, 2015
Urbanization	Proportion of urban population in total population	Haitian Institute of Statistics and Informatics, 2015

Data for most of these variables were extracted from spatially interpolated maps produced by the Demographic and Health Survey (DHS) Program [22]. The maps were freely available as raster files on the DHS Program Spatial Data Repository. The maps were based on a 2016–2017 survey of a nationally representative sample of 13,405 households in Haiti [23]. Using a simple mean approach, datapoints in the maps were aggregated to match the boundaries of each commune using R programming language [24]. Spatial data relative to administrative boundaries and health facilities in Haiti were retrieved from Humanitarian Data Exchange (HDX)–an open access platform managed by the United Nations Office for the Coordination of Humanitarian Affairs [21]. Other data sources included the MSPP and the IHSI.

### Ethical considerations

Since all datasets used in this study were anonymized and aggregated at the commune level, no consent was required. The study was approved by Haiti’s National Bioethics Committee (reference number: 1921–45) and by the University of Nottingham’s School of Medicine Research Ethics Committee (reference number: 267–1903).

### Descriptive analysis

Collected data were examined for consistency by checking for missing, duplicate, and out-of-range values. Frequency distributions were generated for categorical variables. Measures of location (i.e., mean, median) and variation (i.e., standard deviation, range, interquartile range) were calculated for continuous variables. Choropleth maps were developed to illustrate the geographic distribution of the study variables. QGIS [25] was used to process data while the descriptive analysis was performed using the R programming language.

Two variables (DT vaccine stockout and DTP vaccine stockout) were excluded from the analysis due to the large amount of missing data (>10%). Out-of-range values were found for DTP3 vaccine coverage; however, since these values represented <10% of the total number of observations, the variable was included in the analysis. No duplicate values were found in the dataset.

### Spatial autocorrelation and hotspot analysis

Spatial autocorrelation analyses were conducted to investigate the spatial pattern of diphtheria incidence and identify hotspots. The global spatial test Moran’s I was used to quantify the spatial autocorrelation of diphtheria incidence in Haiti. The Moran’s I is an index that measures the extent of spatial autocorrelation in a given dataset using a scale from -1 to +1 [26,27]. A positive Moran’s I suggested positive autocorrelation (i.e., the clustering of communes with similar values). A negative Moran’s I denoted negative autocorrelation (i.e., the clustering of communes with dissimilar values). A Moran’s I close to 0 indicated that values were randomly distributed.

Since the global Moran’s I revealed the overall degree and direction of spatial autocorrelation but not where the clustering of high and low values occurred, local indicators of spatial association (LISA) were also calculated. LISA are a local version of the Moran’s I, in which the level of spatial clustering is assessed around each individual geographical unit (e.g., commune) rather than across the entire study area (e.g., Haiti) [28]. In this study, neighbour relationships were defined using a first-order Queen’s contiguity method, in which only communes that shared common boundaries were considered to be neighbours. If a commune was situated on an island and, thus, did not share borders with the rest of the study area, these were assigned manually to one of the nearest communes on mainland Haiti [29]. The main output of the LISA analysis was a map showing four types of statistically significant spatial autocorrelation [28]: high-high to indicate the clustering of communes with high diphtheria incidence (i.e., the hotspots); low-low to show the clustering of communes with low incidence (i.e., the cold spots); and, low-high and high-low to represent spatial outliers (i.e., low incidence communes surrounded by high incidence communes, and vice versa).

All spatial analyses were conducted in GeoDa 1.12 [30]. The level of significance was set at p<0.05. Significance of spatial tests was evaluated by comparing the observed test results with the expected results under the complete spatial randomness assumption using Markov chain Monte Carlo (MCMC) method based on 999 permutations [31].

### Regression models

To identify the significant correlates of diphtheria incidence, two regression models were built: ordinary least squares (OLS) and geographically weighted regression (GWR). OLS is a global model which presumes that observations are mutually independent and that relations between dependent and independent variables are constant across a study area. When these assumptions are violated, global models are no longer effective. OLS is defined as [32]:

$$Y=\beta_{0}+\beta_{1}X_{1}+\beta_{2}X_{2}+\ldots \beta_{n}X_{n}+\varepsilon ,$$ (1)

where *Y* is the dependent variable, *X* is the independent variable, *β* is the coefficient explaining the strength and type of relationship between *X* and *Y*, and *ε* is the residual (i.e., the difference between observed and predicted values).

In contrast with OLS, GWR is a local model that accounts for spatial heterogeneity by generating a unique equation for every unit of a study area [33,34]. Each equation is calibrated based on their neighbouring units, which are weighted using a decreasing function of distance; in other words, nearby areas hold a greater weight than those farther away. The assumption is that everything is related to everything else, but near things are more related than distant things (i.e., Tobler’s first law of geography) [35]. GWR can be defined as:

$$Y_{i}=\beta_{0i}+\beta_{1i}X_{1i}+\beta_{2i}X_{2i}+\ldots \beta_{ni}X_{ni}+\varepsilon_{i}$$ (2)

in which *i* is the specific location where data on *Y* and *X* are measured.

Independent variables to be included in the two models were identified using a multi-stage process to ensure the absence of multicollinearity, which occurs when independent variables are highly correlated among each other [36]. Firstly, Spearman’s rank correlation was conducted to identify strong correlations (r≥0.7, p≤0.05). If two or more independent variables were highly correlated, the one with the lowest correlation with diphtheria incidence was excluded. Then, the remaining variables were included in the OLS model. Finally, the variance inflation factor (VIF) was calculated to determine the degree of multicollinearity among the independent variables. A VIF≤5 was considered acceptable. Variables that did not have a statistically significant (p>0.1) effect on diphtheria incidence were removed from the model.

The performance of the OLS and GWR models was compared using the adjusted r-squared (R^2^) and the corrected Akaike information criterion (AIC_c_). R^2^ is the coefficient of determination, which indicates the proportion of variance in the dependent variable that is collectively explained by the independent variables [37]. A drawback of *R*^2^ is that it increases with the number of added variables. The adjusted R^2^ is similar to the ordinary R^2^, but it imposes a penalty as superfluous variables are included in the model. AIC_c_ is a modified version of the Akaike information criterion (AIC), a comparative measure of goodness-of-fit that takes into account model complexity [38]. AIC is obtained by the sum of twice the negative log-likelihood and twice the number of parameters in the model. Lower AIC scores are indicative of higher efficiency (i.e., models that explain a greater amount of variation using fewer parameters). AIC_c_ is equivalent to AIC but with a correction for small sample sizes.

Results output from the GWR model were used to create surface maps of the R^2^ values and local coefficients of each independent variable to explore the spatial variation in the relationship between diphtheria incidence and the selected parameters. All regression models and surface maps were developed using the R programming language.

## Results

### Descriptive analysis

From December 2014 to August 2021, 392 confirmed diphtheria cases were recorded in Haiti (**Table 2**). Most of the cases were female (n = 215; 54.8%) and aged ≤14 years old (n = 343; 87.5%). Only 59 cases (15.1%) were reported to be vaccinated against diphtheria, which was defined as having received at least three doses of a diphtheria vaccine.

**Table 2:** Characteristics of confirmed diphtheria cases in Haiti, December 2014 –June 2021.

Characteristics	n (%)
Total confirmed cases	392
Female	215 (54.8)
Male	177 (45.2)
Age (in years)	
<5	84 (21.4)
5–14	259 (66.1)
>14	49 (12.5)
Vaccination status	
n/a*	4 (1.0)
Unknown	209 (53.3)
Unvaccinated	120 (30.6)
Vaccinated	59 (15.1)

* Cases for which information on the vaccination status was not available.

**Fig 1:**
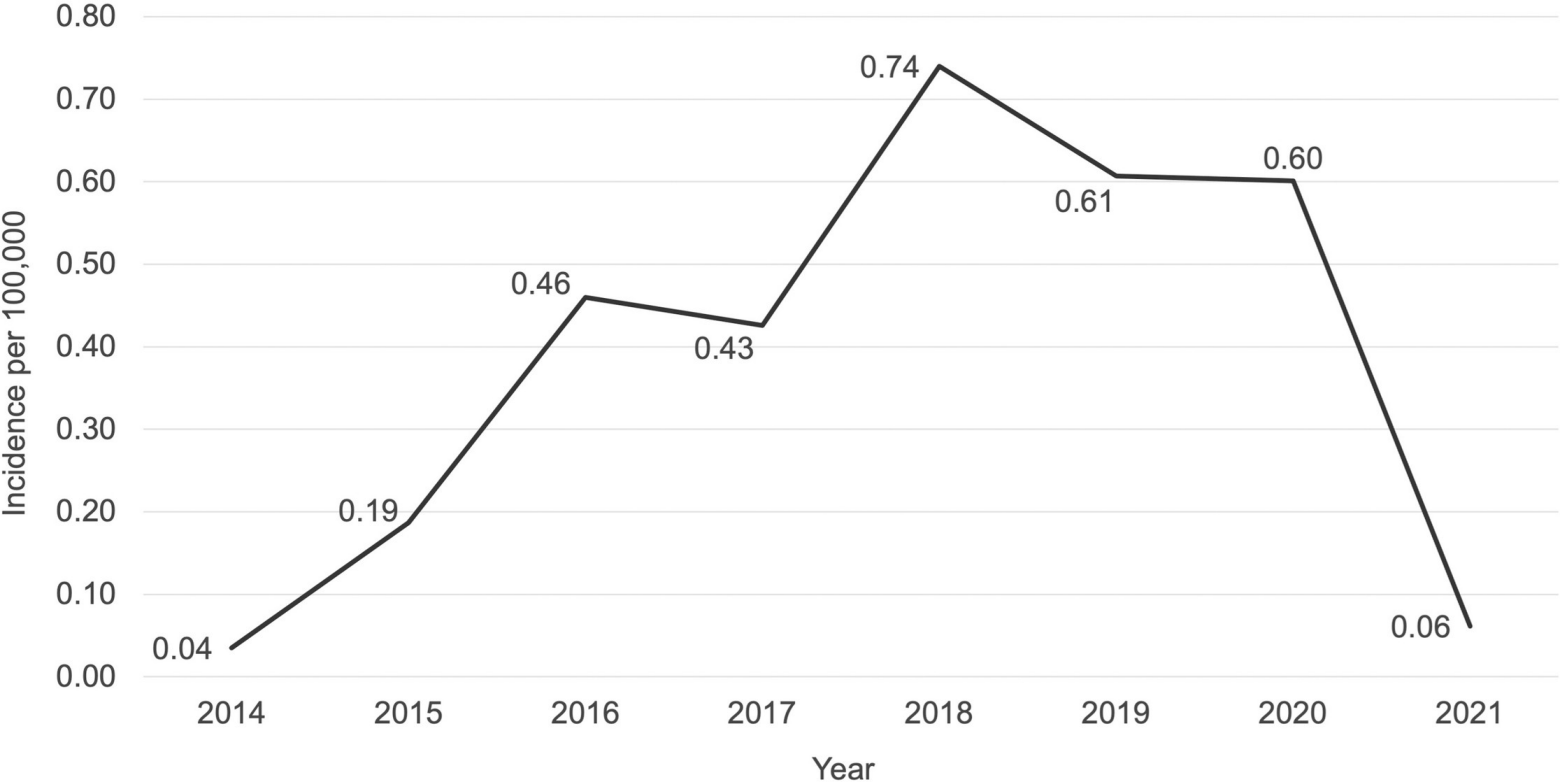
Average annual incidence of confirmed diphtheria cases in Haiti, December 2014 –June 2021.

During the study period, the annual incidence of diphtheria varied greatly, going from 0.04 cases per 100,000 population in 2014 to 0.74 per 100,000 in 2018 (**Fig 1**). This peak was followed by a three-year decline in reported infection rates.

**Fig 2:**
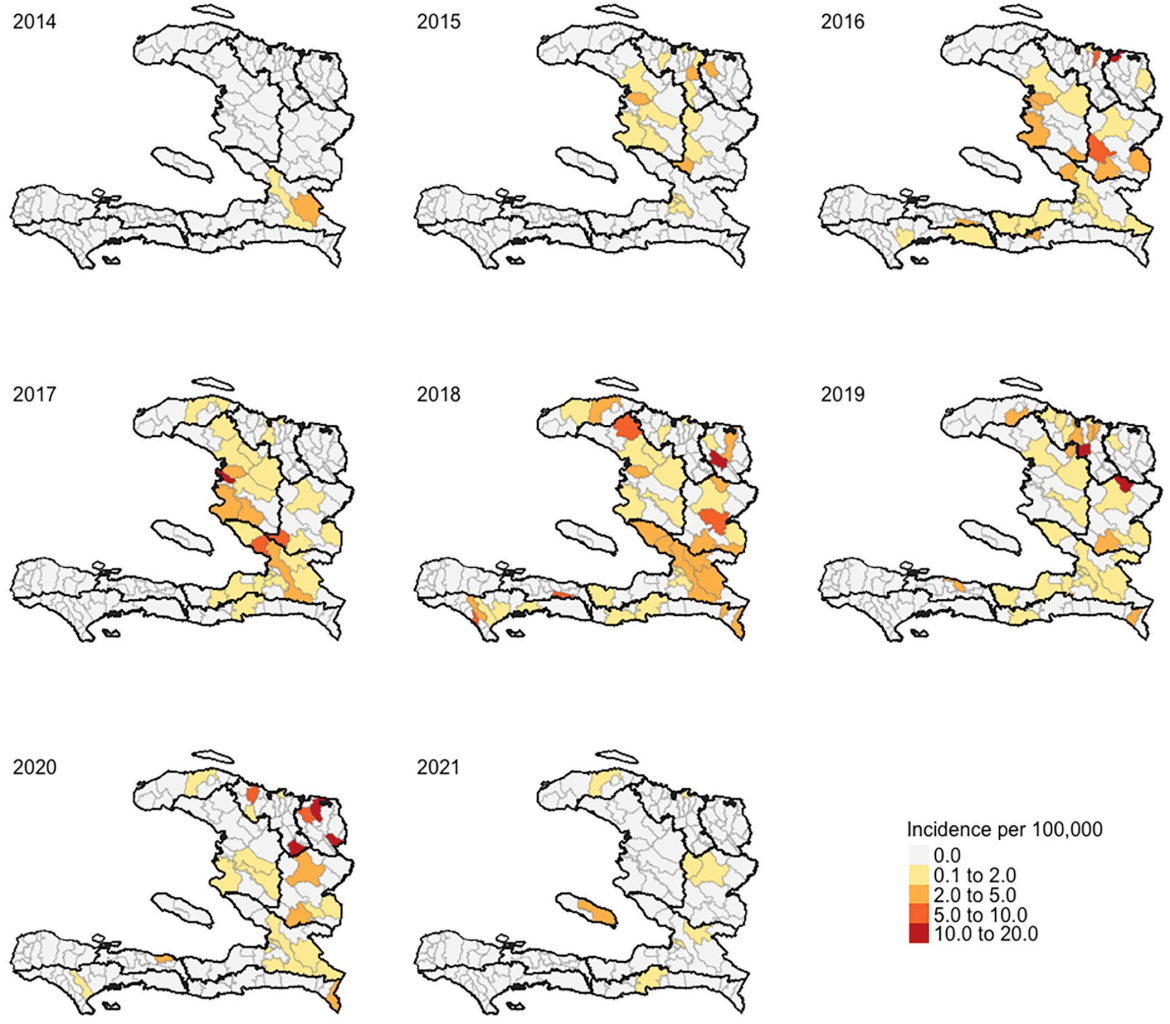
Diphtheria incidence (per 100,000) in Haiti, December 2014 –June 2021.

Information on the commune of origin was not available for two of the 392 cases. As **Fig 2** shows, the outbreak appeared to originate in the Ouest department and to have gradually spread to the rest of the country. Between 2014 and 2015, detection of diphtheria cases remained limited to 21 communes across five departments located in central and northern Haiti. By 2021, cases had been reported in 79 communes, encompassing nine departments. Grand’Anse was the only department to report no confirmed cases throughout the study period. Four departments (i.e., Artibonite, Centre, Nord, and Ouest) accounted for 84% of all confirmed cases. Ouest was the only department to report cases each year.

#### Spatial autocorrelation and hotspot analysis

The global Moran’s I test found modest but statistically significant spatial autocorrelation (I = 0.18, p < 0.001). This suggests that, during the study period, diphtheria incidence was more similar in certain neighbouring communes than would be expected by chance.

**Fig 3:**
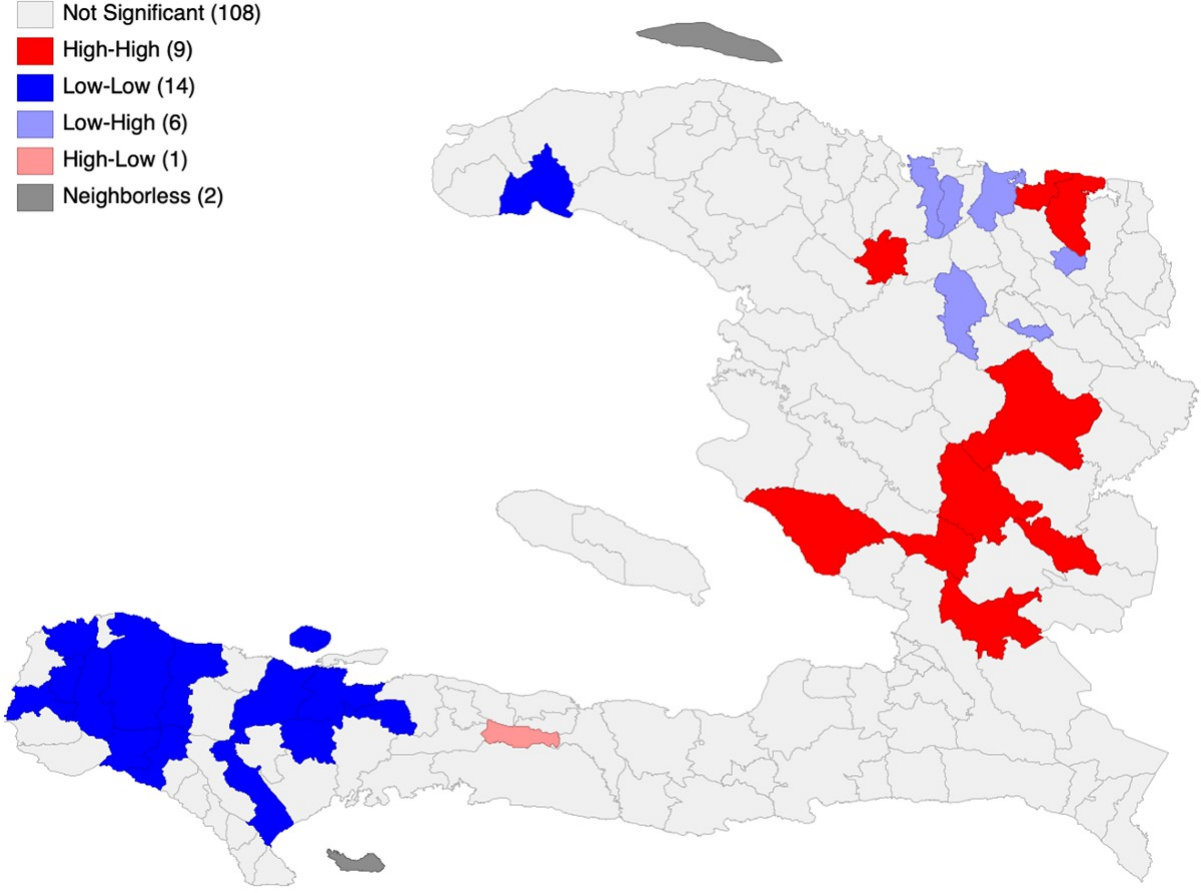
LISA map of average diphtheria incidence (per 100,000), Haiti, December 2014 –June 2021.

The LISA analysis revealed nine communes, home to an estimated 646,346 people (4.7% of the population of Haiti), that can be classified as diphtheria hotspots (**Fig 3**). Furthermore, one high-low commune (i.e., a high incidence commune surrounded by areas of low diphtheria incidence) was found in the Sud department. An estimated 35,139 people (0.3% of the population) live in this high-low commune. Additionally, the analysis identified 14 cold spots and six low-high outliers (i.e., low incidence communes surrounded by areas of high diphtheria incidence). **S1 Appendix** lists the identified areas with spatial dependence.

### Regression models

The Spearman’s rank correlation analysis found that male literacy and female literacy were highly correlated (r = 0.78, p<0.001). Consequently, male literacy was excluded from the pool of independent variables as it did not have a significant correlation with diphtheria incidence (p = 0.18). Low collinearity was observed among the remaining variables (VIF range = 1.18–2.22).

**Table 3** presents the results of the regression analyses. In the final OLS model, health facility density and the degree of urbanization were positively associated with diphtheria incidence. Specifically, for every one-unit increase in health facilities per 100,000 population, the rate of diphtheria cases per 100,000 population was reported to increase by 0.020. Similarly, a one-unit increase in the proportion of population who lives in urban areas led to a 0.009 increase in the rate of diphtheria cases per 100,000. Conversely, a negative association was observed with female literacy. A one-unit increase in female literacy rate was found to decrease the rate of diphtheria cases per 100,000 by 0.030. The adjusted R^2^ for the final OLS model was 0.15, which indicates that the model explains 15% of the variance seen in diphtheria incidence. The R^2^ value suggest a weak model fit and explanation of variance. The AIC_c_ score was 267.13.

**Table 3:** Summary of the OLS ^a^ and GWR ^b^ models.

Parameter	Initial OLS	Final OLS	Final GWR
DTP3 coverage	0.177 (0.488)		
Health facility density	0.015 (0.007) *	0.015 (0.005) **	0.015
Improved water source	0.003 (0.003)		
Female literacy	-0.026 (0.007) ***	-0.024 (0.006) ***	-0.024
No toilet facility	< -0.001 (0.004)		
School density	-0.001 (0.002)		
Population density	< -0.001 (< 0.001)		
Urbanization	0.007 (0.003) **	0.006 (0.002) **	0.006
Adjusted R^2^	0.14	0.15	0.28
AIC_c_	274.88	267.13	261.97

^a^ For the OLS models, estimates correspond to the coefficients and the standard error in parentheses.

^b^ For the GWR model, estimates correspond to the mean coefficients.

* P<0.05

** P<0.01

*** P<0.001.

The GWR model incorporated the same variables as the final OLS model. There was agreement between the OLS and GWR model on the direction of the influence of the selected independent variables on diphtheria incidence. Furthermore, the effect sizes for the independent variables were the same in the two models. However, the GWR model considerably improved model performance and fit compared to the final OLS model, as indicated by the higher adjusted R^2^ value (0.28) and lower AIC_c_ score (261.97). These results suggest that, by accommodating spatial non-stationarity and allowing variables to vary in space, the GWR model is better than the OLS model at explaining the relationship between diphtheria incidence and other factors.

**Fig 4:**
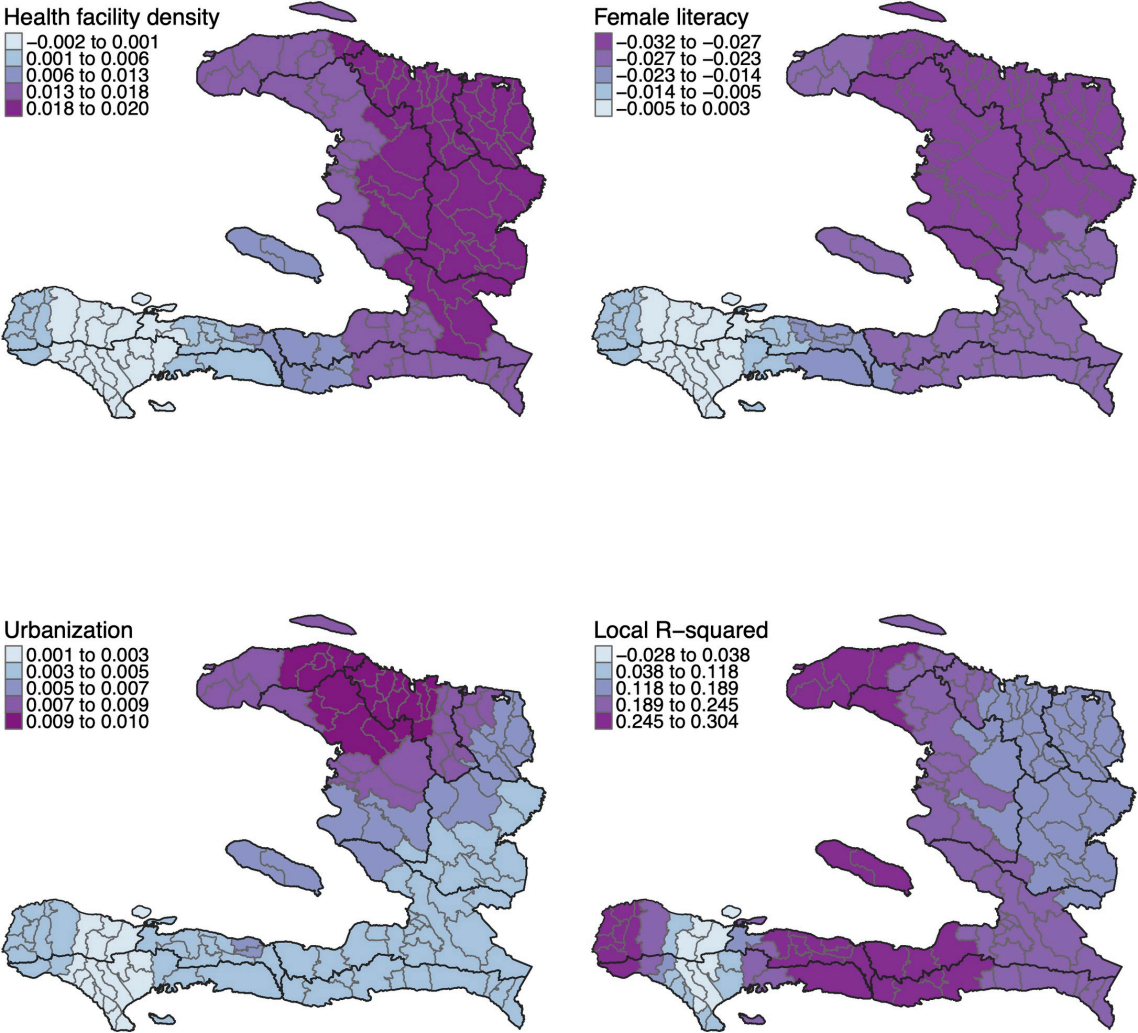
Local regression coefficients and R^2^ values.

**Fig 4** shows the variation in the local coefficient estimates of the GWR model and the R^2^ value for each commune. These maps reveal that the influence of the three independent variables in the model varies considerably across Haiti. The local coefficients of health facility density (range = -0.002–0.020) tended to be higher in the central and northern departments of the country. The largest coefficients for female literacy (range = -0.032– -0.003) were found in Artibonite, parts of Centre and Ouest, as well as in the northern departments. Coefficients for urbanization (range = 0.001–0.010) appeared to be higher in the Nord and Nord’Ouest departments and in the northernmost communes of Artibonite. The map of the local R^2^ values (range = 0.01–0.35) indicates that the level of explanatory power of the GWR model varies significantly throughout the territory, with higher local R^2^ values found in as many as six different departments.

## Discussion

This study has shown that the reported incidence of the disease varied considerably between December 2014 and June 2021, reaching a peak in 2018. The investigation has identified **areas with spatial dependence**, which suggests that certain communes in Haiti may have predisposing factors increasing the risk of diphtheria transmission. This hypothesis is supported by findings from the GWR model, which have demonstrated that at the commune-level 28% of the variability in diphtheria incidence in Haiti could be explained by a combination of three factors: health facility density, the degree of urbanization, and female literacy.

The sharp increase in incidence in the early stages of the outbreak indicates that a large proportion of the population in Haiti was susceptible to diphtheria. This is consistent with the results of Minta *et al*. [39], who found no evidence of long-term protection against the infection (IgG≥1 IU/mL) among a nationally representative sample of 1,146 children aged 5–7 years in Haiti in 2017. There are a few probable explanations for the decrease in incidence after 2018. That year, the MSPP conducted a mass vaccination campaign that saw more than two million children aged 1–14 years receiving at least one dose of a diphtheria vaccine [39,40]. It is reasonable to assume that the campaign contributed to reducing the size of susceptible individuals, ultimately driving down the incidence of the disease. Nevertheless, the decline in incidence may have also been partly a surveillance artifact. Since 2019, there has been a dramatic surge in politically motivated protests and civil unrest, which has been accompanied by high levels of gang-related violence throughout Haiti [41]. This period has also coincided with the emergence of COVID-19 [42]. The two crises have paralyzed the country for long periods of time, making it more difficult for people in need to access medical care and for health authorities to conduct basic surveillance activities, such as case investigation and contact tracing. As a result, several diphtheria cases may have gone undetected, which suggests that available figures likely underestimate the disease’s true spread.

By characterizing the spatial distribution of detected diphtheria incidence, we have shown that the disease has spread widely across Haiti. Nevertheless, substantial heterogeneities in diphtheria incidence exist from one department to another and between communes within the same department. The LISA analysis brought to light a spectrum of diphtheria dynamics that includes several areas with spatial dependence. An estimated 646,346 people (4.7% of the population of Haiti) are living in diphtheria hotspots. Interestingly, some of the identified hotspots are located near the border with the Dominican Republic, which has reported diphtheria cases in recent years [9]. This indicates that close collaboration between the two countries, especially on cross-border surveillance, would be crucial to control the transmission of diphtheria on the Hispaniola island. The hotspots detected in this study could be prioritized for targeted public health interventions, including raising people’s awareness about diphtheria and preventive measures through community health workers, training clinical personnel periodically, and increasing the capacity for laboratory testing. All these interventions have shown promise in the response to other public health issues in Haiti [43–45]. However, given that the full implementation of these measures will require considerable investment and time, vaccination continues to be the most vital tool in the fight against diphtheria.

The associations of diphtheria incidence with health facility density, degree of urbanization, and female literacy were somewhat expected. In areas with a high number of clinics and hospitals, the probability of detecting a diphtheria case is higher than elsewhere because of the increased access to healthcare services [46,47]. Urban areas are generally characterized by overcrowding as well as high population mobility and inter-mixing, all of which increase the opportunities for infectious diseases, like diphtheria, to spread [48,49]. Literate women might comprehend health messages better than illiterate women, which makes them more likely to take protective measures (e.g., vaccination and personal hygiene) for themselves and for their children [50,51]. These findings add to existing evidence that health outcomes are shaped by factors beyond healthcare [52,53].

The coefficient estimates of the GWR model highlighted spatial variations in the relationships between diphtheria incidence and the three independent variables. This suggests that the level of influence of each independent variable on diphtheria incidence might have varied from one commune to another. Gaining these local-level insights simply would have not been possible using global OLS techniques. These findings should be complemented by qualitative studies to understand why and how the interrelationships between diphtheria incidence and the independent variables differ across Haiti. Such investigations might help to better explain the observed differences in diphtheria incidence.

Of note in our results is the lack of association between diphtheria incidence and risk factors related to vaccination, especially given that just 15% of the confirmed cases in this study were reported to be vaccinated against diphtheria. Past research has highlighted several issues related to vaccination coverage measurements, including coverage estimates sometimes exceeding 100%, improbable year-to-year variations, and epidemics in areas reporting high coverage [54]. These issues can be linked to weaknesses in immunization information systems (IIS) and inaccuracies in vaccination coverage denominators. Unfortunately, Haiti faces both problems. A multi-country evaluation from 2009 found major flaws in the national ‬IIS [55]. It is probable that some of those inadequacies are still present today. Furthermore, Haiti’s vaccination coverage estimates are unlikely to be accurate as they are based on population projections–the last official census dates back to 2003 [56]. It is, thus, plausible that inadequate vaccination contributes to the propagation of diphtheria in the country, though this cannot be demonstrated through this study.

A number of limitations may have affected our findings. Although diphtheria is a nationally notifiable disease in Haiti, some underreporting by physicians may still occur for a variety of reasons, including misdiagnosis. Additionally, asymptomatic cases and symptomatic individuals who did not seek medical care may have gone unreported. Consequently, notified cases may not necessarily reflect the actual incidence of diphtheria. Moreover, data for the examined variables were from different time periods, which reduces the reliability of the regression estimates. Furthermore, data on certain risk factors known to correlate with diphtheria were unavailable (e.g., level of wealth, knowledge of diphtheria) [20], impeding further analysis. Additionally, as our models were based on aggregated data, there is a risk of ecological fallacy, which consists in assuming that associations observed at the commune level will necessarily hold at the individual level [57]. Finally, like other analytic methods, GWR has some drawbacks: its spatial weighting function accounts for geographical distance but ignores the attributes of the observations [58]; local multicollinearity may be present in a GWR model, even if the independent variables are not collinear at the global level [59]. Given these limitations, alternative approaches have been proposed, including conditional autoregressive (CAR) models, simultaneous autoregressive (SAR) models, and Bayesian hierarchical models [59,60].

To our knowledge, this is the first study that describes the epidemiology of diphtheria in Haiti using GIS and spatial analysis. The study has shown that GWR is a useful technique for exploratory and descriptive data analysis, which not only improves on the OLS performance but enables the discovery of hidden spatial relationships between variables. This investigation has also demonstrated that between 2014 and 2021 diphtheria exhibited spatial variability in Haiti, with the clustering of high and low incidence areas. The hotspots detected in this analysis could serve as a basis for prioritizing and targeting response activities. The baseline estimates of diphtheria incidence presented in this paper could guide surveillance activities and help track progress in the control of the disease. Further research and continued monitoring of the factors found to be associated with diphtheria incidence could help us better understand the spread of the disease.

## Supporting information

S1 Appendix (DOCX)

## References

[1] Evans AS, Brachman PS. Bacterial infections of humans: epidemiology and control / edited by Alfred S. Evans and Philip S. Brachman. 3rd ed. ed. New York: New York: Plenum Medical Book Co.; 1998.

[2] Centers for Disease Control and Prevention. Manual for the surveillance of vaccine-preventable diseases. Atlanta, GA;: Centers for Disease Control and Prevention;; 2008.

[3] Tiwari TSP. 37—Diphtheria. In: Magill AJ, Hill DR, Solomon T, Ryan ET, editors. Hunter’s Tropical Medicine and Emerging Infectious Disease. 9th ed. London: W.B. Saunders; 2013. p. 402–6.

[4] Blumberg LH, Prieto MA, Diaz JV, Blanco MJ, Valle B, Pla C, et al. The preventable tragedy of diphtheria in the 21st century. Int J Infect Dis. 2018;71:122–3. doi: 10.1016/j.ijid.2018.05.002 29871739

[5] Truelove SA, Keegan LT, Moss WJ, Chaisson LH, Macher E, Azman AS, et al. Clinical and Epidemiological Aspects of Diphtheria:A Systematic Review and Pooled Analysis. Clin Infect Dis. 2019. 10.1093/cid/ciz808 PMC7312233 31425581

[6] World Health Organization. Diphtheria reported cases: World Health Organization,; 2020 [Available from: http://apps.who.int/immunization_monitoring/globalsummary/timeseries/tsincidencediphtheria.html.

[7] European Centre for Disease Prevention and Control. Gap analysis on securing diphtheria diagnostic capacity and diphtheria antitoxin availability in the EU/EEA. Stockholm: European Centre for Disease Prevention and Control,; 2017.

[8] Kupferschmidt K. Life-saving diphtheria drug is running out. Science. 2017;355(6321):118. doi: 10.1126/science.355.6321.118 28082541

[9] Pan American Health Organization / World Health Organization. Epidemiological Update: Diphtheria in Hispaniola. 25 June 2021. Washington, D.C.; 2021.

[10] Clerville J. Diphtheria Outbreak, Haiti, 2014–2017: An Epidemiological Profile and A Case Fatality Rate Trend Analysis. Int J Infect Dis. 2018;73:274–5.

[11] Exavier MM, Paul Hanna M, Muscadin E, Freishstat RJ, Brisma JP, Canarie MF. Diphtheria in Children in Northern Haiti. J Trop Pediatr. 2018. 10.1093/tropej/fmy021 29688558

[12] Juin S. Resurgence of Diphtheria in Haiti: Observations from the National Epidemiologic Surveillance System: 2014–2018. American Journal of Tropical Medicine and Hygiene. 2018;99(4):596–.

[13] Smith CM, Le Comber SC, Fry H, Bull M, Leach S, Hayward A. Spatial methods for infectious disease outbreak investigations: systematic literature review. Eurosurveillance. 2015;20(39):6–26. doi: 10.2807/1560-7917.ES.2015.20.39.30026 26536896

[14] Kirby RS, Delmelle E, Eberth JM. Advances in spatial epidemiology and geographic information systems. Annals of Epidemiology. 2017;27(1):1–9. doi: 10.1016/j.annepidem.2016.12.001 28081893

[15] Podavalenko AP. Estimating the complication risk of epidemic situation with diphtheria in Ukraine. Asian Journal of Epidemiology. 2018;11(1):26–33.

[16] Nailul I, Soenarnatalina S. Analysis of Spatial Data of Diphtheria Disease in East Java Province during the year 2010 and 2011. Buletin Penelitian Sistem Kesehatan. 2015;18(2):211–9.

[17] Quesada GM, Cameron JM Jr, Anderson DE, Kaufert JM. Risk analysis of the 1970 San Antonio diphtheria epidemic. Disasters. 1978;2(4):221–30. doi: 10.1111/j.1467-7717.1978.tb00100.x 20958388

[18] Haitian Institute of Statistics and Informatics. Total population, population aged 18 years and over, estimated households and densities in 2015. In: Directorate of Demographic and Social Statistics, editor. Port-au-Prince: Haitian Institute of Statisitics and Informatics,; 2015.

[19] Graves KA. Haiti: Capstone; 2006.

[20] Ikejezie J, Adebusoye B, Ekezie W, Phalkey R, Langley T, Lewis S. Modifiable risk factors for diphtheria: a systematic review and meta-analysis. Manuscript in preparation. 2022. 10.1016/j.gloepi.2023.100100 PMC10445968 37638375

[21] Humanitarian Data Exchange. Haiti: Humanitarian Data Exchange,; 2019 [Available from: https://data.humdata.org/group/hti.

[22] ICF. The DHS Program Spatial Data Repository. Funded by USAID. 2019 [Available from: spatialdata.dhsprogram.com.

[23] Haitian Institute of Childhood (IHE) HIoSaI, ICF International, Ministry of Public Health and Population (Haiti),. Haiti Demographic and Health Survey 2016–2017. Fairfax, United States of America: ICF International; 2018.

[24] R Development Core Team. R: A language and environment for statistical computing. Vienna: R Foundation for Statistical Computing,.

[25] QGIS Development Team. QGIS Geographic Information System: Open Source Geospatial Foundation Project,; 2019 [Available from: http://qgis.osgeo.org.

[26] Moran PAP. A Test for the Serial Independence of Residuals. Biometrika. 1950;37(1/2):178–81. 15420264

[27] Mitchell A. The ESRI guide to GIS analysis, Volume 2. Environmental Systems Research I, editor. Redlands Calif.: Redlands, Calif.: Environmental Systems Research Institute; 2005.

[28] Anselin L. Local Indicators of Spatial Association—LISA. Geographical Analysis. 1995;27(2):93–115.

[29] Ward MDG, Kristian Skrede Spatial regression models. Gleditsch KS, editor. Thousand Oaks: Thousand Oaks: Sage Publications; 2008.

[30] Anselin L, Syabri I, Kho Y. GeoDa: An Introduction to Spatial Data Analysis. Geographical Analysis. 2006;38(1):5–22.

[31] Ripley BD. Spatial statistics: John Wiley & Sons; 2005.

[32] Stewart Fotheringham A, Charlton M, Brunsdon C. The geography of parameter space: an investigation of spatial non-stationarity. International Journal of Geographical Information Systems. 1996;10(5):605–27.

[33] Brunsdon C, Fotheringham AS, Charlton ME. Geographically Weighted Regression: A Method for Exploring Spatial Nonstationarity. Geographical Analysis. 1996;28(4):281–98.

[34] Fotheringham AS, Rogerson P. The SAGE handbook of spatial analysis [electronic resource] / edited by A. Stewart Fotheringham and Peter A. Rogerson. Los Angeles, [Calif.] London: Los Angeles, Calif. London: SAGE; 2009.

[35] Tobler WR. A Computer Movie Simulating Urban Growth in the Detroit Region. Economic Geography. 1970;46:234–40.

[36] The problem of multicollinearity. In: Allen MP, editor. Understanding Regression Analysis. Boston, MA: Springer US; 1997. p. 176–80.

[37] Forthofer RN. Biostatistics: a guide to design, analysis, and discovery / Ronald N. Forthofer, Eun Sul Lee, Mike Hernandez. 2nd ed. ed. Lee ES, Hernandez M, editors. Burlington, Mass. London: Burlington, Mass. London: Elsevier Academic Press; 2007.

[38] Akaike H. Information Theory and an Extension of the Maximum Likelihood Principle. In: Parzen E, Tanabe K, Kitagawa G, editors. Selected Papers of Hirotugu Akaike. New York, NY: Springer New York; 1998. p. 199–213.

[39] Minta AA, Andre-Alboth J, Childs L, Nace D, Rey-Benito G, Boncy J, et al. Seroprevalence of Measles, Rubella, Tetanus, and Diphtheria Antibodies among Children in Haiti, 2017. Am J Trop Med Hyg. 2020;103(4):1717–25. doi: 10.4269/ajtmh.20-0112 32618256 PMC7543806

[40] Pan American Health Organization / World Health Organization. Haiti launches campaign to vaccinate over 2 million children against diphtheria, with PAHO support Port-au-Prince, Haiti,: Pan American Health Organization / World Health Organization,; 2018 [Available from: https://www.paho.org/en/news/10-4-2018-haiti-launches-campaign-vaccinate-over-2-million-children-against-diphtheria-paho.

[41] United Nations Integrated Office in Haiti. Report of the Secretary-General—S/2020/123. Port-au-Prince, Haiti; 2020 13 February 2020.

[42] World Health Organization. Coronavirus Disease 2019 (COVID-19) Situation Report–61. Geneva, Switzerland; 2020.

[43] Holm M, Burkhartzmeyer H. Implementation of a phased medical educational approach in a developing country. Glob Health Action. 2015;8:29882. doi: 10.3402/gha.v8.29882 26562146 PMC4643178

[44] Louis FJ, Osborne AJ, Elias VJ, Buteau J, Boncy J, Elong A, et al. Specimen Referral Network to Rapidly Scale-Up CD4 Testing: The Hub and Spoke Model for Haiti. J AIDS Clin Res. 2015;6(8). doi: 10.4172/2155-6113.1000488 26900489 PMC4756652

[45] Séraphin MN, Xinguang C, Ayoya MA, Ngnie-Teta I, Boldon E, Mamadoultaibou A, et al. Childhood anemia in Rural Haiti: the potential role of community health workers. Global Health Research and Policy. 2017;2(1):3. doi: 10.1186/s41256-016-0022-7 29202071 PMC5683206

[46] Nelli L, Guelbeogo M, Ferguson HM, Ouattara D, Tiono A, N’Fale S, et al. Distance sampling for epidemiology: an interactive tool for estimating under-reporting of cases from clinic data. International Journal of Health Geographics. 2020;19(1):16. doi: 10.1186/s12942-020-00209-1 32312266 PMC7171748

[47] Hierink F, Okiro EA, Flahault A, Ray N. The winding road to health: A systematic scoping review on the effect of geographical accessibility to health care on infectious diseases in low- and middle-income countries. PLOS ONE. 2021;16(1):e0244921. doi: 10.1371/journal.pone.0244921 33395431 PMC7781385

[48] Saker L, Lee K, Cannito B, Gilmore A, Campbell-Lendrum DH, Research UNWBWSPf, et al. Globalization and infectious diseases: a review of the linkages / Lance Saker… [et al.]. Geneva: World Health Organization; 2004.

[49] Norwegian Institute of Public Health. Urbanization and preparedness for outbreaks with high-impact respiratory pathogens. Oslo, Norway; 2020.

[50] Vikram K, Vanneman R, Desai S. Linkages between maternal education and childhood immunization in India. Social Science & Medicine. 2012;75(2):331–9. doi: 10.1016/j.socscimed.2012.02.043 22531572 PMC3495071

[51] LeVine RA, Rowe ML. Maternal Literacy and Child Health in Less-Developed Countries: Evidence, Processes, and Limitations. 2009;30(4). 10.1097/DBP.0b013e3181b0eeff 19672161

[52] Braveman P, Gottlieb L. The social determinants of health: it’s time to consider the causes of the causes. Public Health Rep. 2014;129 Suppl 2(Suppl 2):19–31. doi: 10.1177/00333549141291S206 24385661 PMC3863696

[53] Marmot M, Wilkinson R. Social Determinants of Health. Oxford: Oxford: Oxford University Press; 2005.

[54] Stashko LA, Gacic-Dobo M, Dumolard LB, Danovaro-Holliday MC. Assessing the quality and accuracy of national immunization program reported target population estimates from 2000 to 2016. PLOS ONE. 2019;14(7):e0216933. doi: 10.1371/journal.pone.0216933 31287824 PMC6615593

[55] Bosch-Capblanch X, Ronveaux O, Doyle V, Remedios V, Bchir A. Accuracy and quality of immunization information systems in forty-one low income countries. Tropical Medicine & International Health. 2009;14(1):2–10. doi: 10.1111/j.1365-3156.2008.02181.x 19152556

[56] The World Bank. International development association project appraisal document on a proposed grant in the amount of sdr 3.8 million (US$5 million equivalent) to the Republic of Haiti for a statistical capacity building project. The World Bank; 2017.

[57] Robinson WS. Ecological Correlations and the Behavior of Individuals. Int J Epidemiol. 2009;38(2):337–41. doi: 10.1093/ije/dyn357 19179346

[58] Shi H, Zhang L, Liu J. A new spatial-attribute weighting function for geographically weighted regression. Canadian journal of forest research. 2006;36(4):996–1005.

[59] Wheeler DC. Diagnostic Tools and a Remedial Method for Collinearity in Geographically Weighted Regression. Environment and planning A. 2007;39(10):2464–81.

[60] Finley AO. Comparing spatially‐varying coefficients models for analysis of ecological data with non‐stationary and anisotropic residual dependence. Methods in ecology and evolution. 2011;2(2):143–54.

