# Supplementary material for: The epidemiology of diphtheria in Haiti, December 2014–June 2021: A spatial modeling analysis
Source: PLoS One. 2022 Aug 22;17(8):e0273398. doi: 10.1371/journal.pone.0273398 (PMC9394811; doi:10.1371/journal.pone.0273398)
Supplement: S1 Appendix — (DOCX) [file pone.0273398.s001.docx]

**APPENDIX 1**

**Areas with spatial dependence in Haiti identified in the LISA analysis**

| Type of spatial dependence | n | Department | n | Commune |
| --- | --- | --- | --- | --- |
| High-high | 9 | Centre | 4 | Boucan Carre, Hinche, Lascahobas, Saut d’Eau |
|  |  | Nord-Est | 2 | Caracol, Terrier Rouge |
|  |  | Ouest | 2 | Arcahaie, Thomazeau |
|  |  | Artibonite | 1 | Marmelade |
| Low-low | 14 | Grand’Anse | 7 | Abricots, Anse d’Hainault, Chambellan, Jérémie, Moron, Pestel, Roseaux |
|  |  | Sud | 4 | Chardonnieres, Les Anglais, Maniche, Torbeck |
|  |  | Nippes | 2 | Baraderes, Plaisance du Sud |
|  |  | Nord-Ouest | 1 | Baie de Henne |
| Low-high | 6 | Nord | 5 | La Victoire, Limonde, Milot, Plaine du Nord, Saint-Raphael |
|  |  | Nord-Est | 1 | Perches |
| High-low | 1 | Sud | 1 | Fonds des Negres |
